# V483A: an emerging mutation hotspot of SARS-CoV-2

**DOI:** 10.2217/fvl-2020-0384

**Published:** 2021-06-22

**Authors:** Omar Ashwaq, Pratibha Manickavasagam, SK Manirul Haque

**Affiliations:** 1 ^1^Department of Chemical & Process Engineering Technology, Jubail Industrial College, Jubail Industrial City, 31961, Saudi Arabia; 2 ^2^Department of Biotechnology, Indian Institute of Technology Hyderabad, Kandi, Telangana, India

**Keywords:** high transmission, infectivity, spike protein, substitution mutation, V483A, virus–host cell interaction

## Abstract

One of the many mutations that have occurred in the viral genome is the V483A mutation, which is a part of the receptor-binding motif present in the S1 domain of the spike protein. V483A mutant virus is popular in North America with 36 cases so far and frequently occurring in recent days. This review compares the wild-type and the V483A mutants to analyze certain factors like the interaction between the virus and host-cell interface, binding affinity, stability, partition energy, hydrophobicity, occurrence rate and transmissibility. This information can be of monumental importance in vaccine and drug development since the mutants can become resistant to the vaccines and monoclonal antibodies.

Human society is presently witnessing a new lockdown world that has emerged out of the progressing COVID-19 crisis. It seems as if the virulent nature of the SARS-CoV-2 has increased over the past few months, and its infection is continuously sweeping the world at a relentless infection rate. Researchers and scientists worldwide are scrambling their resources and struggling to find a suitable solution to curtail this deadly virus’s further spread. The viral architecture of the SARS-CoV-2 contains four different structural proteins, namely nucleocapsid (N), membrane (M), envelope (E) and spike (S) protein [1,2]. The S protein consists of two subunits S1 and S2, with a furin cleavage site S`. The S1 domain of the S protein contains the receptor-binding domain (RBD), and the S2 domain has the fusion peptide domain. The spike protein of the virus includes approximately 1273 amino acids, where the S1 domain starts from 13 to 685 amino acid, and the S2 domain is from 686 to 1273 amino acid (Figure 1) [3]. The RBD of S1 binds to the human ACE2 receptor, which is present on the epithelial cells. The human ACE2 receptor present on the epithelial cells binds to the RBD of S1 protein through the receptor-binding motif (RBM). RBD contains a highly conserved core region made of β sheets and the highly variable RBM region [4]. RBD is located between amino acid residues 319–541, and the RBM is located between residues 437–508 of S-protein (Figure 1) [3].

**Figure 1.**
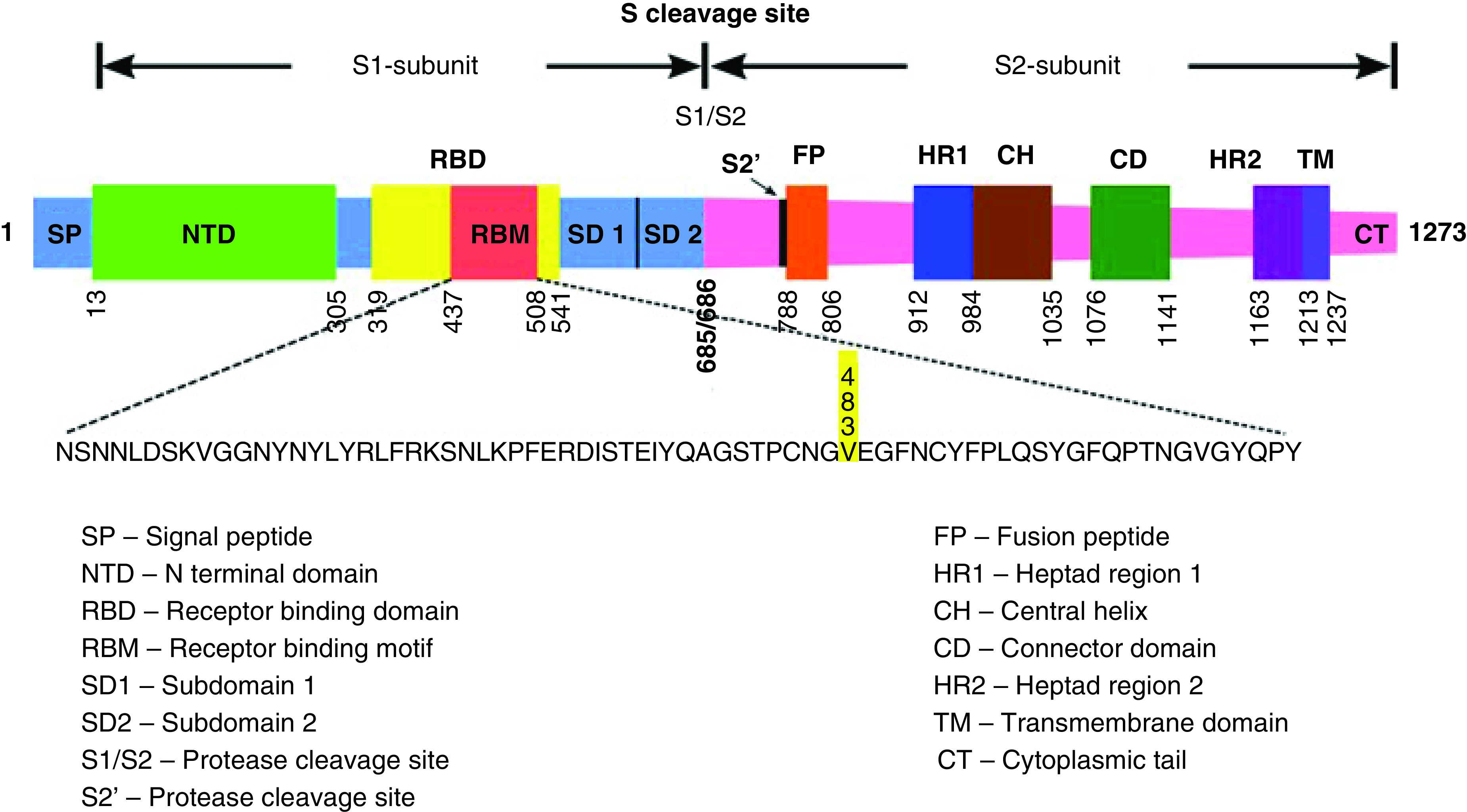
Schematic representation of the domains in the S protein of SARS-CoV-2.

The fusion peptides present in the S2 subunit fuses the ACE2 receptor and enable a successful entry into the host cell. The presence of a furin cleavage site at the S protein differentiates it from other beta coronaviruses. The ubiquitous expression of proteases in the host cell cleaves the S protein and aids in structural rearrangement. It then successfully fuses the virus with the host cell, thus infiltrating the host cell [5].

Mutations in SARS-CoV-2 are causing another area of concern during the pandemic. It is found that the SARS-CoV-2 is mutating more rapidly, and to date, over 82,062 mutations have occurred, and it is still counting [6]. The human immune system’s intervention is one reason for such rapid mutation in the viral genome. Due to such rapid mutation, there is high variability in the genome, thereby posing a challenge for scientists to find a suitable drug or vaccine. It is feared that the mutations can bring sequence variation and structural changes in the virus, which can lower the effect of vaccines on these viruses by evading the host immune system through antibody escape [7]. During this outbreak, many ongoing studies are focused on vaccine designing, drug repurposing, understanding the virus’s pathogenicity, etc. The study of single amino acid mutation analysis is done very extensively. The mutation study on S protein helps understand the virus’s virulence, antibody escape variants and cellular tropism. Especially mutation study of the residues at the interface of the RBM and ACE2 has a significant role in potential pharmacophores for developing therapeutic drugs [7]. In recent studies, the S protein has been looked upon as a potential immunogen because it is seen as the most accessible part of the virus. This protein is majorly responsible for the high infection rate of the virus [8]. Among many other mutations, the RBM mutations are seen as a hot spot, leading to a higher infection rate.

In this review, we have singled out one such mutation, the V483A, which lies within the RBM of the RBD. It is a critical amino acid residue in the RBM region of the spike glycoprotein, where the valine (Val) at position 483 has changed to alanine (Ala) (Figure 2), making the viral genome a unique mutant strain [9]. V483A is a few mutations that can change the protein secondary structure and relative solvent accessibility in the RBM region [10]. The RBM makes the contacts between the SARS-CoV-2 and the human ACE2 receptor acting as the core binding site. The schematic representation of domains and motifs and the V483 amino acid residue position in the SARS-CoV-2 spike protein are shown in Figure 1.

**Figure 2.**
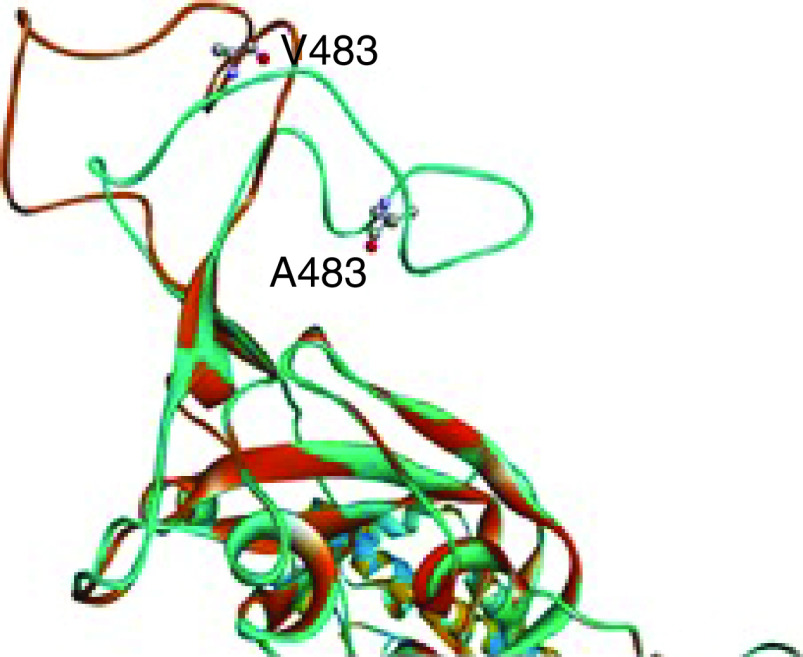
Loop configuration-wild-type structure is in orange, and mutant is in blue.

Furthermore, the RNA replication rate causes the virus to mutate faster, evading host immunity, thereby posing strong drug resistance. This mutagenic capability of the virus has become the leading cause of its evolution and genomic variation [11]. This review aims to comprise all currently available information of the V483A mutant type and its characteristics compared with the wild-type strain.

## The transition of nucleotide induces the substitution mutation

Due to the transition from thymine (uracil) to cytosine at the genome position 23,010, the new clade or substrain of the virus has evolved. Thus, Val at the 483rd position of S protein got substituted for Ala [12]. This nonsynonymous (i.e., amino acid altering) substitution mutation increases in this novel coronavirus. The nonsynonymous functional transformation in retroviruses will bring functional constraints to the protein, and the mutation is a part of natural selection [13]. Many ssRNA viruses show a stringent (Darwinian) selection to bring about positive changes in the virus, favoring its transmission [14]. The nonsynonymous mutation is increasing at a higher RBD rate of the spike protein [15]. Apart from the primary (amino acid change) and secondary (loop structure) changes observed at the RBM of S protein due to V483A mutation, there are few gains in functions like higher solvent accessibility, tighter binding of the mutant protein to the receptor and aid in antibody escape mechanism that will be discussed in this review. Therefore, the V483A mutation can become one of the virus’s favorable mutations and increase the transmission rate [16].

## Structure analysis of mutant protein

**Figure 3.**
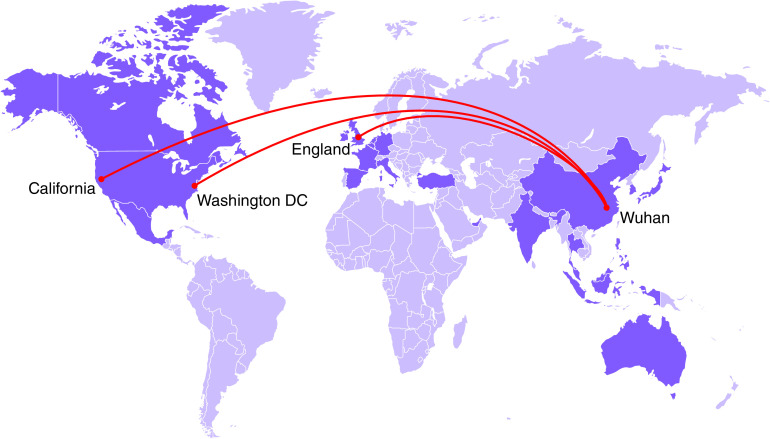
The wild strain from China, Wuhan got mutated and transmitted to various parts of the world as a V483A variant.

In V483A mutation, the amino acid Val, which is hydrophobic, present at the 483rd position of the spike protein at the RBD of S1 got substituted as hydrophobic Ala in some of the sequences. This mutation is present in the RBD loop region of S1 [13]. This mutation is reported as one of the frequent mutations. The mutation occurrence from March 2020 till date is reported as 36 (Figure 3) [8], and this mutant is found to be more occurrent in the USA [17].

**Figure 4.**
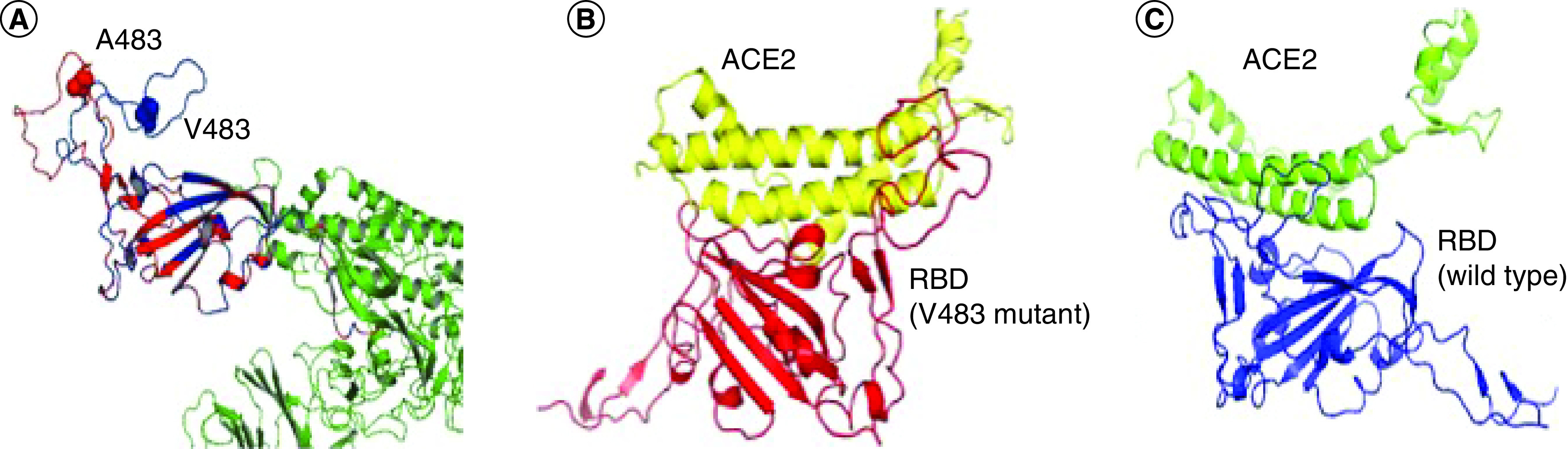
Spike protein of SARS-CoV-2. **(A)** The RBD of S1 domain of the wild-type is in blue and mutant in red. **(B)** The docked pose of mutant V483A RBD interacting with human ACE2 receptor helix 1. **(C)** The docked pose of wild-type RBD to the human ACE2 receptor helix 1 [11]. RBD: Receptor-binding domain.

The overall secondary structure upon binding to the ACE2 receptor for both wild-type and mutant did not change hugely (Figure 4). But upon binding, there is a 1% increase in the RBD loop region (α-helix and βsheet) of the mutant protein [16]. The V483A mutation site does not form direct contact with the ACE2 receptor, but it is on the same face of the RBD that includes the binding interface with the ACE2 [18]. Along with V483A, other mutations were reported at the 483rd position of the RBD where Val is getting substituted to other amino acids such as phenylalanine (Phe), isoleucine (Ile), proline (Pro), aspartic acid (Asp), arginine (Arg) and lysine (Lys) in very low occurrence rate [19,20].

## Binding energy comparison

**Figure 5.**
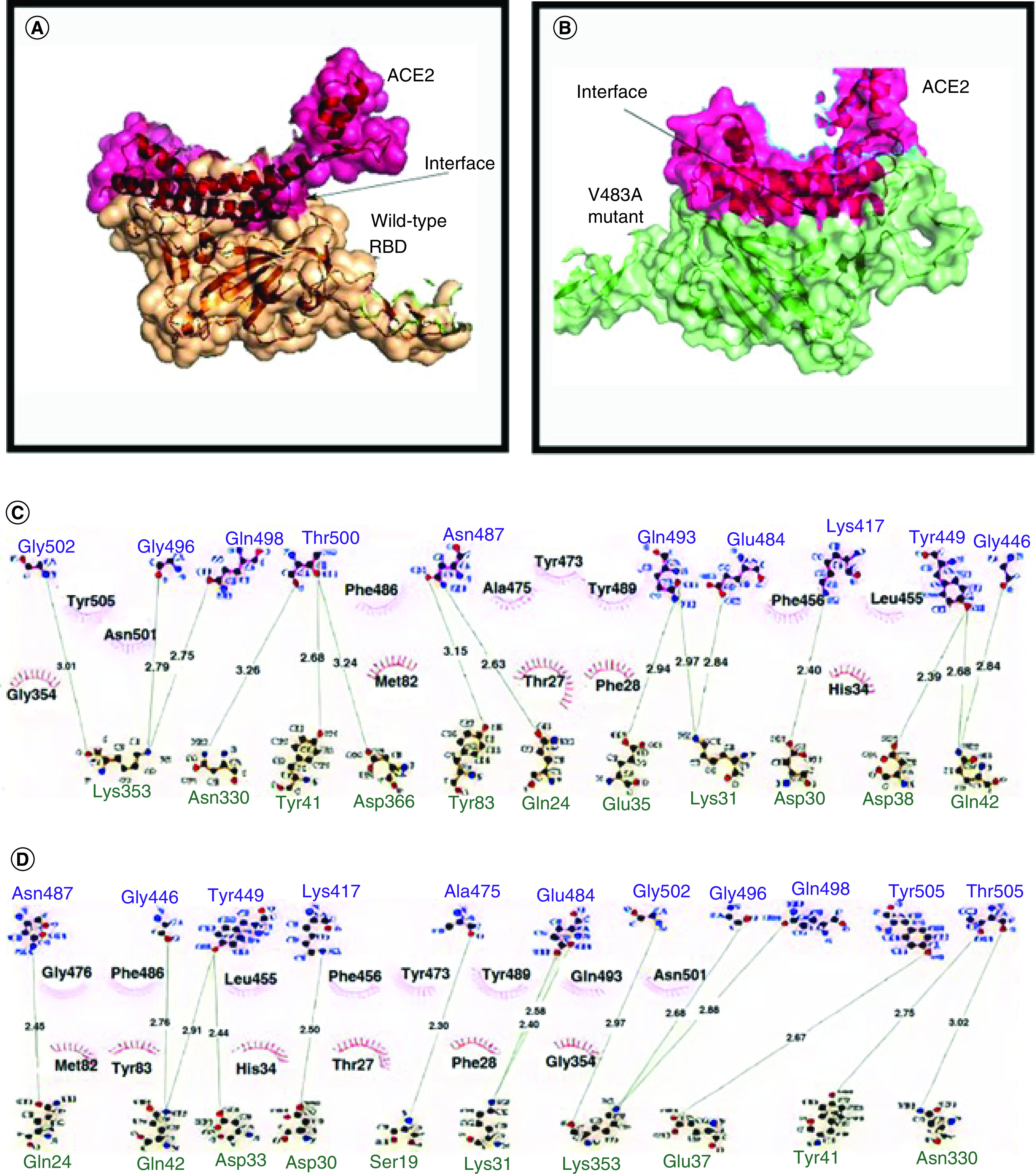
Interaction diagram for receptor-binding domain and ACE2 complex. **(A)** 3D representation of the interaction between the receptor-binding domain of S1-domain with the human ACE2 receptor helix-1 chain, a wild-type S protein. **(B)** Mutant V483A. **(C & D)** Represents the ball and stick model of interacting amino acids of wild-type and V483A mutant, respectively, where aa in blue belongs to S protein, and green belongs to human ACE2 receptor [22].

The free binding energy (ΔG) of the wild-type S protein to the human ACE2 receptor is -14.1 kcal/mol, and for the V483A mutant, it is -15.2 kcal/mol [9]. Therefore, the change in the binding energy after mutation is ΔΔG, calculated by calculating the difference between the free binding energy ΔG of wild-type from the mutant [21]. For V483A, the ΔΔG is found to be (ΔΔG = ΔG wild-type - ΔG mutant) +1.1 kcal/mol. The ΔΔG value is positive, so the mutation increases the binding affinity, and hence the mutant can become more stable and more infective in the future [21]. The short-range Coulombic interaction energy between the RBD of wild-type and the human ACE2 receptor in a dynamic environment is -2.307 × 10^5^ kcal/mol, and for V483A mutant is -2.320 × 10^5^ kcal/mol. Thus, the mutant protein can bind much better and can be stable during the interaction with the ACE2 receptor compared with the wild-type [22]. During the interaction between two proteins, hydrogen bonds are formed to make a protein–protein complex. Also, the number of hydrogen bonds formed between the complex may significantly stabilize the complex [4]. The number of H-bonds involved with wild-type and the V483A mutant is estimated by 20 ns simulation using molecular dynamics (MD) studies. From the molecular dynamics simulation studies, it is observed that on average, 7.283 ± 1.568 H-bonds were formed between the ACE2 receptor and the V483A mutant strain, whereas in the tabulated H-bond data from the molecular docking study, it is reported that around 5.651 bonds were formed between the ACE2 receptor and the wild-type S protein [22]. Therefore, the outcome predicts that the mutated model includes a highly stable complex with ACE2 receptor rather than its wild-type. The root also determines the protein–protein complex’s stability to root mean square deviation (RMSD) analysis. The docking studies have been performed between the RBD region of the spike protein and the ACE2 receptor. The RMSD values are on average 3.6 ± 0.57 and 3 ± 0.43 Å for wild-type and mutated residue at V483, respectively [22]. Figure 5 represents the 3D model of the wild-type and the mutant S protein RBD binding to the human ACE2 receptor and the H-bond details in the ball and stick representation. Table 1 points to the H-bond interaction between the S-protein and the receptor with the bond distance [22].

**Table 1.** The amino acids of S protein involved in interacting with the human ACE2 receptor-A chain of helix 1 in the wild-type and mutant are tabulated below.

H-bond interaction between hACE2 receptor and amino acid residues		Hydrophobic interaction between S protein and hACE2 receptor	
Wild-type (reference)	Mutated (V483A)	Wild-type (reference)	Mutated (V483A)
Tyr41-**Thr500** (2.68)	Tyr41-**Thr500** (2.75)	Gly354	Met82
Gln24-**Asn487** (2.63)	Gln24-**Asn487** (2.45)	**Tyr505**	Tyr83
Lys31-**Gln493** (2.97)	Lys31-**Gln493** (2.58)	**Asn501**	**Gly476**
Lys31-**Glu484** (2.84)	Lys31-**Glu484** (2.40)	Met82	**Phe486**
Lys353–**Gly502** (3.01)	Lys353-**Gly502** (2.97)	**Phe486**	His34
Lys353–**Gly496** (2.79)	Lys353-**Gly496** (2.68)	Thr27	**Leu455**
Lys353-**Gln498** (2.75)	Lys353-**Gly498** (2.88)	Phe28	Thr27
Asn330-**Thr500** (3.26)	Asn330-**Thr500** (3.02)	**Tyr473**	**Phe456**
Gln42-**Tyr449** (2.68)	Gln42-**Gly446** (2.76)	**Ala473**	**Tyr473**
Gln42-**Gly446** (2.84)	Gln42-**Tyr449** (2.91)	**Tyr489**	Phe28
Asp38-**Tyr449** (2.39)	Asp38-**Tyr449** (2.44)	His34	**Tyr489**
Asp30-**Lys47** (2.40)	Asp30-**Lys417** (2.50)	**Phe456**	Gly354
Asp355-**Thr500** (3.24)	Ser19-**Ala475** (2.30)	**Leu455**	**Gln493**
Tyr83-**Asn487** (3.15)	Glu37-**Tyr505** (2.67)		**Asn501**
Glu35-**Gln493** (2.94)			

Amino acids of S protein are in black; human ACE2 receptors in boldface

Data taken from [22].

## Role of mutation in increasing the stability

Though the V483A mutation is at the RBM of the S1 protein, the amino acid is not making direct contact with the ACE2 receptor. Compared with SARS-CoV-1, this mutant protein binds four- to tenfold tightly to the receptor [15]. The V483A mutation with higher frequency indicates that this mutation may favor SARS-CoV-2 by natural selection may cause this virus to be more infectious. The binding affinity change is positive, indicating the mutation will help make tighter interactions with the receptor [17]. Cryogenic electron microscopy studies show that this type of nonsynonymous single nucleotide variations may affect the strength of transmission of the virus [23]. The dynamic studies on a structural basis due to this mutation revealed that the binding surface of the RBD to ACE2 is having predominantly random coil conformation, and it lacks structural rigidity. To get the firm scaffold, beta-sheet structures are provided by the 510–524 amino acids of S protein [24]. The V483 mutant site in the RBD of S1 is close to Q24 of ACE2. V483 is more than 10 Å away from the Q24, one of the interacting amino acids of ACE2. It could affect the receptor binding of SARS-CoV-2 by indirectly altering the RBM of the S protein’s loop region, leading to more stability [25]. The V483A mutant is exposed to solvents as they present on the RBD surface, and the loop region may stick out into the solvent. Thus, this mutation may not directly impact receptor binding or stability. Still, it lowers the hydrophobic surface and reduces the nonspecific stickiness of this loop region and may affect antibody binding [25]. The Kyte–Doolittle hydropathy index value for the V483A mutant is -2.4, where the negative value indicates the loop region’s hydrophilic nature. It is known that Val hydrophobic index (4.2) is higher than the Ala hydrophobic index (1.2) [15]. Results of dynamic study for around 300 ns show that this variant is stable throughout the simulation [7]. From an evolutionary perspective, this mutation may further evolve to be an even more dangerous sub-strain to humans [7]. Other than H-bond formation, nonbonded interactions are formed between the ACE2 receptor and the S protein. These nonbonded interactions may increase the coordination number and increase the binding between the protein–protein complex [26].

## Other amino acid substitution at V483

In the case of V483F mutation, the amino acid Val, which is hydrophobic, is substituted by bulky hydrophobic amino acid Phe, which might influence the glycosylation efficiency nearby amino acid N343 or the positioning of sugars [25]. Other mutations occur at this hotspot, where it is replaced by other amino acids such as proline (Pro), aspartic acid (Asp), lysine (Lys) with a low occurrence rate. Their maximum binding energy difference (ΔΔG) is 3.162 and 0.05 kcal/mol for Pro and Asp acid, respectively. This positive binding energy indicates that these amino acid substitutions can also lead to tighter S protein binding to the receptor. For Lys, the minimum ΔΔG is reported to be -0.851 kcal/mol [20]. Thus, these mutations can also have the potential to emerge as one of the infectious strains (Table 2).

**Table 2.** Overall summary of all the mutations at the 483rd position of V483 of receptor-binding motif in the S1 domain.

SNP position	Nucleotide base substitution	Amino acid substitution	Occurrences (n)	Variants (n)	Clade	Ref.
23010	T to C (transition)	V483A	36	A, F, I, P, D, R, K	S84	[8,19,20,27]

## Immune evasion by mutant

Neutralizing antibodies evoked by either natural infection or vaccination is the beginning of building the populace’s adaptive immunity against SARS-CoV-2 [28]. Passively administering antibodies as a recombinant protein or convalescent plasma is an effective therapeutic and prophylactic measure that can be taken against its infection [29]. The emergence of antibody-resistant variants of this virus hindering the therapy can be brought under control by combining antibodies toward neutralizing epitopes [30].

Studies show that the neutralizing antibody response to infection is critical for creating an effective and durable vaccine [31]. Investigations on the infectivity and reactivity of the V483A variants showed that they were resistant to some neutralizing antibodies. These findings could be of value in the development of vaccine and therapeutic antibodies [8]. A change in the amino acid residue of the RBM of the spike protein can give rise to significant changes in the functionality, infectivity, transmission and interactions of the virus with neutralizing antibodies [32]. The neutralizing antibodies bind to the virus and counteract its effect on the host system [33]. Analysis of the antigenicity of V483A mutant using monoclonal antibodies (mAbs) revealed that V483A became resistant to X593 and P2B-2F6 mAbs. These two monoclonal neutralizing antibody works better for wild-type strain, but its activity is tenfold decreased sensitivity in the mutant strain compared with the wild-type strain [8].

Antibody studies conducted using the antibody 5A6 immunoglobulin-G, which has the superior neutralization capacity with many SARS-CoV-2 mutant strains, including the D614G strain, failed to neutralize the V483A mutant strain. Although the antibody 5A6 IgG had a high occupancy on the viral surface and had bivalent binding capacity binding to both the ‘up’ and ‘down’ positions of the RBD–ACE2 interaction surface, it showed a fourfold reduction in binding avidity to the V483A mutant strain. So it is recommended to administer the antibody 3D11 along with 5A6 IgG to compensate for this failure against the V483A strain [34].

## Scope of vaccine design

Epitope analysis of the V483A mutant strain proved that 13 effective B-cell epitopes significantly advanced the mutant antigenicity compared with the wild-type strain they were 62∼75, 487∼492, 210∼221, 181∼186, 342∼353, 363∼377, 617∼628, 405∼418, 405∼413, 379∼389, 442∼447, 458∼463 and 698∼709. Although these epitopes account for a very small proportion of the population, precautions should be carried out against any antigen escape induced by genetic variation during vaccination [35]. One of the *in silico* study using NetMHC4 software binding affinity between the epitope of S protein to class I MHC using the most frequent HLA is predicted. As well, the software expected 9-mer (e.g., GAEGFNCYF epitope) in which the MHC I can bind effectively to the mutant strain. Positive affinity and varied solvent accessibility will create negative impacts on the vaccine and diagnostic test development. In a recent study, the multi-epitope vaccine designed using DeepVacPred software effectively binds for mutant strain [36].

Although carefully selected therapeutic cocktails will offer greater resistance to SARS-CoV-2 escape, this identified mAbs will significantly help in the preclinical evaluation and development of immune therapeutics to be used against COVID-19 in humans [37]. Since mutations bring about changes only in the spike protein structure without any differences in the ACE2 receptor moiety, it is predicted that vaccines developed to bring around humans’ immunogenicity in fighting the virus cannot be affected by, except if there are any aggressive mutations. The V483A has not been reported as an aggressive mutation, although it is one of the most important mutations after the D614G mutation [21]. In any case, exploring the complete nature of the virus along with each of its mutant strains has always been of paramount importance in designing an effective vaccine and for meaningful therapeutic treatments [7]. A list of protein-based vaccines currently undergoing clinical trial has been given in the table below (Table 3).

**Table 3.** List of vaccines under clinical trials for vaccine development of COVID-19.

Vaccine candidate	Vaccine platform	Developer/manufacturer/country	Expected outcomes/results
ChAdOx1-S (AZD1222)	Nonreplicating viral vector	University of Oxford/AstraZeneca/UK	Enhance the immune response against the spike protein of SARS-CoV-2, which will restrict the entry of the virus to a human cell and prevent the infection
LNP-encapsulated mRNA (mRNA-1273)	RNA	Moderna/NIAID/USA	Block spike protein binding ability with ACE2, as well stopped its consequences and proliferation
Adenovirus type 5 vector (Ad5-nCoV)	Nonreplicating viral vector	CanSino Biological Inc./Beijing Institute of Biotechnology/China	It can neutralize RBD-specific ELISA antibody response to control the deadly virus
Adjuvanted recombinant protein (RBD-Dimer)	Protein subunit	Anhui Zhifei Longcom Biopharmaceutical/Institute of Microbiology, Chinese Academy of Sciences/China	The RBD is essential for immune response. Therefore it is an attractive target vaccine, and RBD-dimer restricts binding with receptors and controls its interference
DNA plasmid vaccine with electroporation (INO-4800)	DNA	Inovio Pharmaceuticals/International Vaccine Institute/South Korea	It can block the host cell’s spike protein and ACE2 receptor by neutralizing SARS-CoV-2 infection and can work against mutant variant D614
Ad26COVS1	Nonreplicating viral vector	Janssen Pharmaceutical Companies/USA and Belgium	Effectively neutralize the antibody and enhanced immune response against SARS-CoV-2 glycoprotein and stop interaction between the host cell’s glycoprotein and ACE2 receptor
RBD based	Protein subunit	Kentucky Bioprocessing, Inc/USA	Initiate antibodies to prevent binding of the subunit (S1/S2) with the receptor and later regulate the membrane fusion to restrain the virus infection
Native like trimeric subunit spike protein vaccine (SCB-2019)	Protein subunit	Clover Biopharmaceuticals Inc./GSK/Dynavax/Australia	
Recombinant spike protein with Advax™ adjuvant	Protein subunit	Vaxine Pty Ltd/Medytox/Australia	
Molecular clamp stabilized spike protein with MF59 adjuvant	Protein subunit	University of Queensland/CSL/Seqirus/Australia	
S-2P protein + CpG 1018	Protein subunit	Medigen Vaccine Biologics Corporation/NIAID/Dynavax/USA	
Full-length recombinant SARS CoV-2 glycoprotein nanoparticle vaccine adjuvanted with matrix M	Protein subunit	Novavax/USA	Highly immunogenic response with specific antibodies that can deactivate the spike protein’s binding capability with receptor present in the human cell and neutralize the antibodies of SARS-CoV-2 wild-type virus and restrict its domain activity

RBD: Receptor-binding domain.

Data taken from [38].

## Conclusion

The V483A mutation represents one of the major emerging mutations of the current COVID-19 pandemic crisis. We found that significant attention has been given by various researchers globally to this particular variant. Data from high-quality research works were coordinated and critically reviewed in all sections of this review. Evidence from different researchers worldwide shows that the next emerging mutation after D614G can severely enhance the infection rate. V483A is not directly related to the virus–host cell interaction, but it can improve the protein–protein complex’s binding stability and binding capacity. We observed that the V483A mutation was first observed in the North American region. Its occurrence is now predominantly increasing in its population and spreading toward the European and Asian region. It is also assumed that this mutation can be one of the key factors for the USA’s higher death rate.

Furthermore, we have highlighted all possible angles and evidence to help researchers get a clear picture of this SARS-CoV-2 variant and investigate potential therapies for its neutralization. We believe the current circumstances justify the prioritization of such mutation studies. There are sufficient insight and a rationale that needs to be suggested to scientists on who the world depends on the inception of a vaccine that can spell this deadly virus’s end.

## Future perspective

Many emerging mutations of the SARS-CoV-2 virus shows us that the virus is yet to find its stable form. Mutation studies on the coronavirus pave the way for researchers to investigate on potential therapies and vaccinations for their effective neutralization. Compiling critical reviews on well-coordinated data from high-quality research works on such mutations helps harness a culture of targeted research on such global pandemics. Significant attention needs to be given by researchers globally to every variant of the SARS-CoV-2 for better understanding the nature of the virus. This review has highlighted all possible evidence that the V483A mutation at the RBM of SARS-CoV-2 may increase the virus’s transmission rate. Reviews with sufficient insight into such mutations may help the scientific community design better vaccines that may curtail the deadly virus’s spread.

**Executive summary**

**Structural analysis of virus & host cell**

- Spike (S) protein is one of the major structural proteins of the SARS-CoV-2 virus and comprises two domains, namely the S1 and S2. The receptor-binding domain (RBD), a part of the S protein, contains two regions: the highly conserved and highly variable regions. The highly variable region is the receptor-binding motif (RBM).

**Virus–host cell interaction & nucleotide residues**

- The interaction between the viruses with the host cell receptor (ACE2) happens via the RBM. Examining the residues present at the RBM helps understand the pharmacophore and thereby helps in therapeutic drug development.

**Binding energy changes**

- The V483 residue is not directly involved in the interaction with the ACE2 receptor, but it is at the same interface. Therefore, the free binding energy change is positive (1.1 kcal/mol), stating that this positive change may favor the mutant to bind more tightly to the ACE2 receptor than the wild-type.

**Stability increases due to mutation**

- The number of H-bonds formed between the ACE2 receptor and the mutant is slightly higher than wild-type RBM. Also, binding studies show that the mutant S protein is binding four- to tenfold higher than the wild-type S protein to the ACE2 receptor.
- The V483A mutant favors higher solvent accessibility because the mutation is present at the surface of the RBD and is exposed to the solvent region.

**V483 amino acid substitutions**

- Many data were reported on mutations occurring at the RBM of S protein. One such emerging mutation at the RBM is V483A. This substitutional mutation (Val to Ala change at 483rd position) is a nonsynonymous functional mutation observed at the RBD loop region. Other than V483A mutation, some other substitutional mutations like V483F, V483I, V483P, V483D, V483R and V483K at a lower frequency rate.

**Immune evasion**

- The antigenicity study reveals that the V483A mutant can neutralize antibodies like X593, P2B-2F6, etc., and attain immune evasion.

**Vaccine & therapeutic treatments**

- Understanding the nature of the virus and its mutant strains is extremely important in designing an effective vaccine and developing meaningful therapeutic treatments.
